# Characterization and Physical and Biological Properties of Tissue Conditioner Incorporated with *Carum copticum L*.

**DOI:** 10.1155/2021/5577760

**Published:** 2021-08-12

**Authors:** Maryam Hejazi, Zahra Zareshahrabadi, Sepideh Ashayeri, Mohammad Jamal Saharkhiz, Aida Iraji, Mohsen Alishahi, Kamiar Zomorodian

**Affiliations:** ^1^Department of Prosthodontics, School of Dentistry, Shiraz University of Medical Sciences, Shiraz, Iran; ^2^Department of Parasitology and Mycology, School of Medicine, Shiraz University of Medical Sciences, Shiraz, Iran; ^3^Prosthodontics Research Center, School of Dentistry, Shiraz University of Medical Sciences, Shiraz, Iran; ^4^Department of Horticultural Sciences, Faculty of Agriculture, Shiraz University, Shiraz, Iran; ^5^Stem Cells Technology Research Center, Shiraz University of Medical Sciences, Shiraz, Iran; ^6^Central Research Laboratory, Shiraz University of Medical Sciences, Shiraz, Iran; ^7^School of Chemical and Petroleum Engineering, Shiraz University, Shiraz, Iran; ^8^Basic Sciences in Infectious Diseases Research Center, Shiraz University of Medical Sciences, Shiraz, Iran

## Abstract

**Aim:**

One of the main problems in dentistry is the injury caused by the long-term application of an ill-fitting denture. The existence of multiple microorganisms along with the susceptibility of the tissue conditioners to colonize them can lead to denture stomatitis. This study is aimed at developing a tissue conditioner incorporated with *Carum copticum L.* (*C. copticum L.*) for the effective treatment of these injuries.

**Materials and Methods:**

The *Carum copticum L.* essential oil composition was determined by gas chromatography-mass (GC-mass) spectrometry. The antimicrobial activity of the essential oil against the standard strains of bacterial and fungal species was determined by broth microdilution methods as suggested by the Clinical and Laboratory Standards Institute (CLSI). The physical and chemical properties of the prepared tissue conditioner were investigated by viscoelasticity, FTIR assays, and the release study performed. Furthermore, the antibiofilm activity of the *Carum copticum L.* essential oil-loaded tissue conditioner was evaluated by using the XTT reduction assay and scanning electron microscopy (SEM).

**Results:**

The main component of the essential oil is thymol, which possesses high antimicrobial activity. The broth microdilution assay showed that the essential oil has broad activity as the minimum inhibitory concentration was in the range of 32-128 *μ*g mL^−1^. The viscoelasticity test showed that the essential oil significantly diminished the viscoelastic modulus on the first day. The FTIR test showed that *Carum copticum L.* essential oil was preserved as an independent component in the tissue conditioner. The release study showed that the essential oil was released in 3 days following a sustained release and with an ultimate cumulative release of 81%. Finally, the *Carum copticum L*. essential oil exhibited significant activity in the inhibition of microbial biofilm formation in a dose-dependent manner. Indeed, the lowest and highest amounts of biofilm formation on the tissue conditioner disks are exhibited in the *Streptococcus salivarius* and *Candida albicans* by up to 22.4% and 71.4% at the 64 *μ*g mL^−1^ concentration of *C. copticum L.* with a statistically significant difference (*P* < 0.05).

**Conclusion:**

The obtained results showed that the *Carum copticum L.* essential oil-loaded tissue conditioner possessed suitable physical, biological, and release properties for use as a novel treatment for denture stomatitis.

## 1. Introduction

One of the main problems in dentistry is the injury caused by the long-term application of an ill-fitting denture [1]. Besides the mechanical deformation of the basal seat mucosa, the space between the denture and mucosa tissue can be susceptible to the accumulation of the microorganism and denture stomatitis [2, 3]. To treat these injuries, denture liner materials are being used. Soft liner materials are classified into two groups of long-term soft denture liners and tissue conditioners [4].

Tissue conditioners are usually used as a temporary relining material to prevent the transfer of masticatory load and evenly distribute the external stresses on the mucosa of the basal seat [5]. In this manner, tissue conditioners possess broad applications in treatments of traumatized soft tissues, mucosal lesions, and surgical wounds [6]. One of the main problems of the tissue conditioners is their susceptibility to the colonization of microorganisms due to their water solubility and degradability, which can result in the exacerbation of denture stomatitis [7]. To overcome this difficulty, various antifungal drugs such as polyenes, azoles, antibiotics, and antiseptics have been prescribed [8]. Since in the case of denture stomatitis microorganisms usually stay in the site of the mucosa, topical delivery of these drugs can better target the infection. In this approach, incorporation of the drugs into the tissue conditioner can lead to a lower cost, simultaneous treatment of the injured mucosa and the infection, and a more convenient method due to reducing the procedural steps.

In this regard, Falah-Tafti et al. investigated the incorporation of nystatin and fluconazole into a commercial tissue conditioner and showed that the nystatin-incorporated tissues completely inhibited the attachment and colonization of *Candida albicans* [9]. In another study, Radnai et al. incorporated chlorhexidine digluconate and miconazole into a tissue conditioner and study its growth inhibition activity against *C. albicans*. Their results indicated that incorporation of miconazole led to a dose-related inhibitory effect on candidal growth [10].

The main problems in applying these drugs are their high toxicity and side effects as well as the potential development of drug resistance in species. Besides, these drugs are usually effective towards the fungal species, whereas denture stomatitis can be composed of different microorganisms. One of the main approaches to overcome these difficulties is the incorporation of herbal essential oils (EOs) and probiotics instead of antifungal and antibiotic drugs [11, 12]. They can possess high antimicrobial effectivity as well as low toxicity due to their organic nature. The other advantage of these EOs is their broad activity against bacterial and fungal species. In the previous studies, tea tree EO and lemongrass EO are incorporated in tissue conditioners and show comparable antifungal activity with fluconazole [13, 14]. *Carum copticum L.* (*C. copticum L.*) belongs to the Apiaceae plant family and is used for the treatment of gastrointestinal, cardiovascular, and respiratory system diseases [15]. Due to the existence of thymol as the main component in its structure, *C. copticum L.* EO has showed desirable antifungal and antibacterial effects as it is stated that phenolic compounds interfere with the cell membrane to change pH and ion homeostasis [16].

This study is aimed at assessing the characterization and physical and biological properties of *C. copticum L*. to develop a tissue conditioner and prosper a simultaneous treatment of the injured mucosa and the denture stomatitis.

## 2. Materials and Methods

### 2.1. Preparation of the *C. copticum L.* EO

Hydrodistillation is known as a conventional and traditional method for the extraction of bioactive compounds, mainly essential oils. To extract the essential oil, 100 g of *C. copticum L.* seeds was taken in a round-bottomed flask and grounded using an electorally grinding mill; then, 800 mL of distilled water was added [17]. The contents were thoroughly mixed, kept at room temperature overnight, and then subjected to hydrodistillation for 4 h, at the boiling range of water and atmospheric pressure, using a glass Clevenger type apparatus according to the method recommended by the European Pharmacopoeia [2]. Finally, the evaporated EO with water vapor was collected in a condenser. The resulting EO was dried over anhydrous sodium sulfate and stored at 4°C after filtration before gas chromatography and gas chromatography-mass spectrometry (GC-MS) analysis.

#### 2.1.1. Analysis of the EO with Gas Chromatography-Mass Spectrometry (GC-MS)

The composition of the EO was determined by conducting GC-MS analysis on a ThermoQuest-Finnigan instrument equipped with a DB-5 column (60 m × 0.25 mm, 0.25 *μ*m film thickness). The oven temperature was programmed to increase from 50°C to 240°C at a rate of 4°C/min and eventually held for 10 min; the temperature of the transfer line and injector was 250°C, and the selected carrier gas was helium at a flow rate of 1.1 mL min^−1^. The quadrupole mass spectrometer was scanned over the 35-600 amu with an ionization current of 150 mA and an ionization voltage of 70 eV. Linear retention indices for all components were determined by coinjection of the samples with a solution containing homologous series of C8-C22 n-alkanes and comparing them and their mass spectra with those of authentic samples or with available library data of the GC-MS system reported in Wiley library [18].

### 2.2. Microbial Strains

The bacterial species of *Streptococcus mutans* (ATCC 35668), *Streptococcus sobrinus* (ATCC 27607), *Streptococcus sanguinis* (ATCC 10556), *Streptococcus salivarius* (ATCC 9222), *Staphylococcus aureus* (ATCC 25923), and *Enterococcus faecalis* (ATCC 51299), as well as the fungal species of *Candida albicans* (ATCC 10261) and *Candida dubliniensis* (CBS 8500), were used for the antimicrobial and antibiofilm investigations. The bacterial species were cultured in Brain Heart Infusion (BHI) and incubated at 37°C for 24 h. The fungal species were also cultured in Sabouraud dextrose agar (SDA) and incubated at 30°C for 24 h.

#### 2.2.1. Antimicrobial Activity of the *C. copticum L.* EO

The minimum inhibitory concentrations (MICs) of EO against microorganisms were investigated by conducting broth microdilution according to the Clinical and Laboratory Standards Institute (CLSI) broth microdilution protocol, with minor modification [19–21]. First, the stock inoculum of the bacterial and fungal species was suspended in appropriate broth media, and the turbidity of the cells was spectrophotometrically adjusted to 0.5 McFarland scale (this yielded stock suspension of 1-5 × 10^6^ CFU/mL for yeasts and 1-1.5 × 10^8^ cells/mL for bacteria). To evaluate the antibacterial activity, serial dilutions of the EO (0.25-128 *μ*g mL^−1^) were prepared using Muller-Hinton broth (MHB) media in a 96-cell microtiter plate. Similarly, the serial dilutions of the essential oil were prepared in RPMI-1640 media buffered with 3-(N-morpholino) propane sulfonic acid (MOPS) buffer for the antifungal investigation. Then, 100 *μ*L of microbial inoculum with 0.5 McFarland concentrations was added to each well of the plate and incubated for 24-48 h at 37°C and 30°C for bacteria and yeast, respectively. In addition, 200 *μ*L of media without any microorganism and EO was placed in the first column of the microplate as the negative control. Besides, 200 *μ*L of inoculums and media without any EO was added to the last column as the growth control (positive controls). The lowest concentration of the *C. copticum L*. that inhibits any viable growth was defined as MIC and evaluated by comparing the optical density of each well to the control one. Reference antibacterial and antifungal agents of ampicillin (AMP; Sigma-Aldrich) and fluconazole (FLU; Sigma-Aldrich), respectively, were obtained from their relevant manufacturers and dissolved in phosphate buffer solution (ampicillin, pH: 8.0; 0.1 mol/mL) and in water (fluconazole) and were used as quality controls. Each experiment was conducted in triplicate.

### 2.3. Mechanical Properties of the Tissue Conditioner

The GC Soft-Liner (GC Corporation, Tokyo, Japan) was used as a tissue conditioner and was prepared at four concentrations of 0, 16, 32, and 64 *μ*g mL^−1^ of the EO by dissolving the powder and EO in the liquid. The specimens stored in 200 mL of distilled water for 1, 3, and 7 days at 37°C each were investigated; a stress relaxation test was conducted using the Santam universal testing machine (Iran) on these specimens at a crosshead speed of 20 inches/minute, to evaluate and statistically compare their elastic modulus.

### 2.4. Biofilm Formation Assays

#### 2.4.1. Biofilm Preparation and Growth

A total of 96 tissue conditioner disks were prepared, and they were divided into four groups (*n* = 24) according to the concentration of EO (0, 16, 32, and 64 *μ*g mL^−1^) incorporated. Then, within a group, three soft denture liner disks were assigned to each strain. The mentioned standard strains of the bacterial and fungal species were cultured on BHI and SDA plates, respectively. After 48 h, one loop of the colonies was transferred to 20 mL broth media in 250 mL Erlenmeyer flasks and incubated overnight in an orbital shaker (100 rpm) at 30°C under aerobic conditions. The microbial cells were harvested and washed twice in sterile phosphate-buffered saline (PBS) (0.8% (*w*/*v*), NaCl (Merck); 0.02% (*w*/*v*), KH_2_PO_4_ (Merck); 0.31% (*w*/*v*), Na_2_HPO_4_+12H_2_O (Merck); and 0.02% (*w*/*v*), KCl (PanReac); pH 7.4). Then, the bacterial and fungal strains were resuspended in MHB and RPMI-1640, respectively, and cell densities were adjusted to 1.0 × 10^6^ cells/mL after counting with a hemocytometer [22, 23].

500 *μ*L of the resulting bacterial and fungal strain suspensions was added to each well of a 24-well tissue culture plate (Corning, St. Louis, MO, USA), which contained the tissue conditioner disks in different concentrations of the EO. In addition, media with *Candida* and bacteria strains but without the tissue conditioners with EOs are considered positive controls. Then, the plates were incubated for 48 h at 37°C. All experiments were performed in triplicate [23].

#### 2.4.2. Quantitative Measurement of Biofilm Inhibition

The metabolic activity of microbial biofilms was calculated using a colorimetric assay. Biofilm formation was performed, by using a 2,3-bis(2-methoxy-4-nitro-5-sulfo-phenyl)-2H-tetrazolium-5-carbox-anilide (XTT) (Sigma, St. Louis, MO, USA) reduction assay as an indicator of cell viability and proliferation which was prepared in Ringers lactate (0.5 mg mL^−1^). The solution was filter-sterilized (0.22 *μ*m pore size) and then stored at −70°C. Prior to each assay, XTT stock solution was mixed with menadione sodium bisulfite (10 mM, Sigma Chemical Co., St. Louis, USA). After 48 h of incubation, the tissue conditioner disks were transferred to a new tissue culture plate and washed twice with sterile PBS. In the following, 500 *μ*L aliquot of XTT/menadione was added to each well of 24-well plates. The plates were incubated at 37°C in a dark room (3 h). Finally, the colorimetric changes were measured at 570 nm by using a microplate reader (BMG Labtech, Berlin, Germany). The cell viability percent was calculated as follows: cell viability percent = (absorbance of test well/absorbance of control well) × 100.

#### 2.4.3. Qualitative Observation of the Biofilm Formation

Scanning electron microscopy (SEM) was used to observe the biofilm that had formed on the surfaces of the tissue conditioner disks. To examine the ultrastructural nature of *Candida* and bacterial strain biofilms grown, tissue conditioner disks were fixed in 2.5% glutaraldehyde in 0.1 M phosphate buffer (pH 7.2) at 4°C for 1 h. After being washed in buffer, the samples were postfixed in 1% osmium tetroxide in the same buffer for 30 min. The samples were dehydrated in graded concentrations of ethanol and critical point-dried in CO_2_ (Polaron Critical Point Dryer). They were coated with colloidal gold (Balzers SCD 050 Sputter Coater, Baltic, Liechtenstein) and viewed under a Leo 435 VP SEM (Oxford Instruments, Oxford, UK) at 15 kV. The SEM images were taken from the tissue conditioner disks to evaluate the biofilm formation.

### 2.5. Fourier-Transform Infrared Spectroscopy (FTIR)

To determine the functional groups of EO and tissue conditioner with and without the EO, FTIR spectroscopy (Spectrum RXI, Perkin Elmer, USA) was executed at the range of 400-4000 cm^−1^ employing KBr pellets at a controlled ambient condition [24].

### 2.6. Release Study

The releasing profile of the EO from the tissue conditioner with a concentration of 64 *μ*g mL^−1^ of EO was investigated. The prepared tissue conditioner was placed in 10 mL of PBS with a pH of 7.2 and subjected to constant stirring at a maintained temperature of 37°C. At different time intervals, 1 mL samples were withdrawn from the solution and refilled with an equal volume of fresh buffer. The concentration of each sample was evaluated using an ultraviolet-visible spectrophotometer with the aid of the EO calibration curve. The experiments were conducted in triplicate.

### 2.7. Statistical Analysis of Data

Descriptive statistics were used to describe the microbial biofilm formation values for the tissue conditioner disks. The quantitative data were presented as the mean and standard deviations. A one-way ANOVA test was used to compare the biofilm formation of the four microbial strains between the different concentrations of EO incorporated in tissue conditioner disks. The *P* value ≤ 0.05 was considered significant.

## 3. Results

### 3.1. GC-MS Investigation of *C. copticum L*. EO

Table 1 tabulates the components of the *C. copticum L*. EO used in this study. As this table suggests, the main component of the EO is thymol as it comprised about 46% of the oil. The other main components are p-cymene (28%) and *γ*-terpinene (24%). These results showed that the *C. copticum L.* EO of this study is thymol-based, which is a suitable result considering the potent antibacterial, antifungal, and antioxidant activities of thymol [25].

### 3.2. Antimicrobial Activity of the *C. copticum L.* EO

Table 2 presents the MIC values of the EO towards the studied bacterial and fungal species. The results show that the EO possesses potent activities against tested bacterial and fungal species as the MIC values are between 32 and 128 *μ*g mL^−1^. The MICs of the EO against microbial species strains affirm the suitability of this EO for incorporation in tissue conditioners in this study.

### 3.3. Mechanical Properties of the Tissue Conditioner

**Figure 1:**
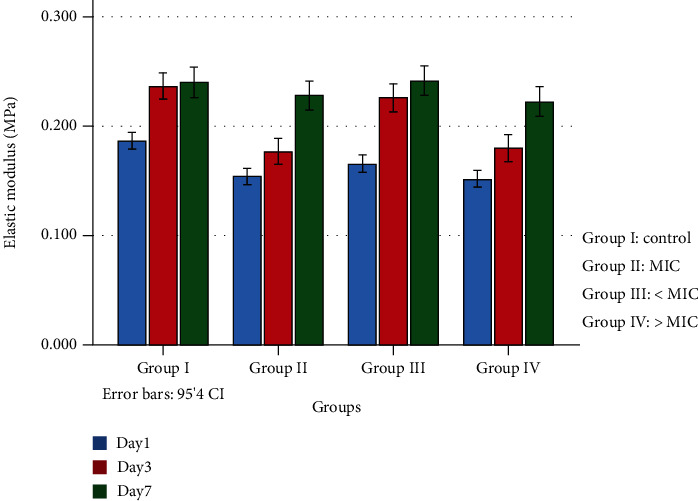
Estimated marginal means and correspondent 95% confidence intervals (CIs) of the elastic modulus of each group at different times.

Figure 1 shows the elastic modulus of the EO at different times and essential oil concentration. As this figure suggests, the elastic modulus of the tissue conditioners decreased by increasing the EO concentration. This figure also shows that the elastic modulus increases over time, and the highest elastic modulus was found to be on day 7 for the same EO concentration. The statistical analysis showed that on day 1, the elastic modulus of the tissue conditioner without EO is significantly higher than the other ones (16, 32, and 64 of the EO) (*P* < 0.05). On day 3, this difference is significant between the tissue conditioner without the EO and the ones with 32 and 64 *μ*g mL^−1^ (*P* < 0.05), whereas on day 7 this difference is insignificant in all groups (*P* > 0.05).

### 3.4. Biofilm Formation of Microorganisms

The mean, standard deviation (SD), and vitality percentage of the microorganisms attached to tissue conditioner disks with different concentrations of EO are shown in Table 3. As are shown in this table, the results indicated that *C. copticum L.* EO exhibited significant activity in inhibition of microbial biofilm formation in a dose-dependent manner, as reflected by lower absorbance reading when compared with the untreated control. Indeed, the lowest and highest amounts of biofilm formation on the tissue conditioner disks are exhibited in the *S. salivarius* and *C. albicans* by up to 22.4% and 71.4% at the 64 *μ*g mL^−1^ concentration of *C. copticum L*. EO, respectively. Generally, biofilm formation of bacterial species strains in the presence of *C. copticum L.* EO was significantly lower than fungi species strains with a statistically significant difference (*P* < 0.05).

**Figure 2:**
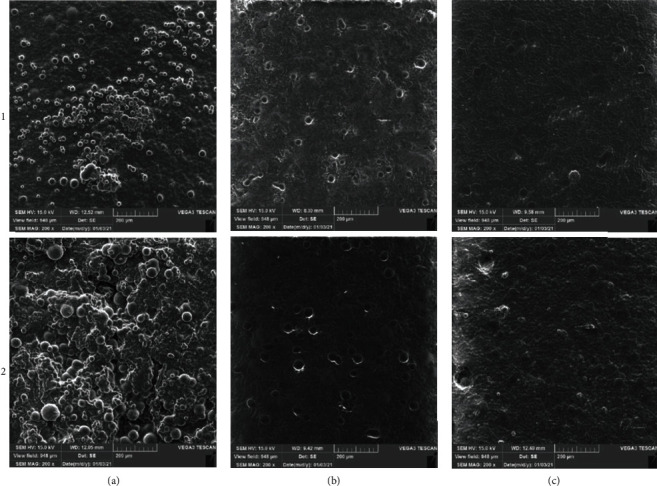
SEM images of the biofilm formation of (1) *S. mutans* (ATCC 35668) and (2) *C. albicans* (ATCC 10261): (a) growth control without EO, tissue conditioner with different *C. copticum L*. EO concentrations, (b) 32 *μ*g mL^−1^, and (c) 64 *μ*g mL^−1^.

The SEM images of biofilm formation of *S. mutans* (ATCC 35668) and *C. albicans* (ATCC 10261) towards tissue conditioner with different concentrations of EO are depicted in Figure 2. As this figure shows, biofilm forms on the surface of the tissue conditioner without EO. According to this figure, by the incorporation of the EO and increasing its concentration into the tissue conditioner, the biofilm formation noticeably decreased.

### 3.5. Fourier-Transform Infrared Spectroscopy (FTIR)

**Figure 3:**
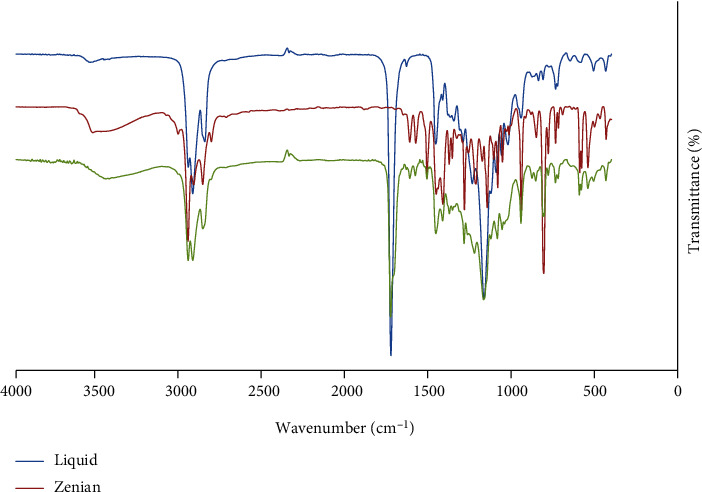
FTIR spectra: (a) tissue conditioner without EO, (b) *C. copticum L*. EO, and (c) tissue conditioner incorporated with *C. copticum L.* EO.

Figure 3 shows the FTIR spectra of the EO and the tissue conditioner with and without the EO. The tissue conditioner of this study consists of poly(ethyl methacrylate), poly(butyl methacrylate), and dibutyl phthalate [26], in which their corresponding characterization peaks are present in Figure 3(a). This spectrum shows a sharp peak at 1731 cm^−1^, which is attributed to the C=O bond of the carbonyl group of poly(ethyl methacrylate) and poly(butyl methacrylate) and their interaction [27]. The triple peaks at 2858, 2933, and 2965 cm^−1^ are assigned to the asymmetrical and symmetrical stretching of C-H bonds of the ethylene group in poly(ethyl methacrylate) and poly(butyl methacrylate) [27, 28]. The characterization peak of dibutyl phthalate is also exhibited at 1164 cm^−1^, which is attributed to C-O stretching [29].

The FTIR spectrum of the *C. copticum L*. EO is also presented in Figure 3(b). This spectrum shows a broad peak from 3300 to 3600 cm^−1^, which is assigned to stretching of the O-H stretching in thymol [30]. The peak at 2866 cm^−1^ is also due to asymmetrical and symmetrical C-H stretching in thymol and p-cymene [30]. The peak at 1585 cm^−1^ is attributed to the stretching of the C-C bonds in the aromatic group [31]. The peaks at 1415 to 1466 cm^−1^ are also due to the asymmetrical and symmetrical bending vibrations of the methyl group [32].

The FTIR spectrum of the EO-loaded tissue conditioner is also shown in Figure 3(c). The spectrum showed the tissue conditioner's peaks at 1732 cm^−1^ (C=O), 1164 cm^−1^ (C-O), and 2859 cm^−1^ and 2969 cm^−1^ (C-H). Comparing the FTIR spectra of tissue conditioners with and without the EO, the EO-loaded one clearly shows a broad characterization peak of *C. copticum L*. EO between 3300 and 3600 cm^−1^. There is also another extra peak at 1585 cm^−1^ due to the presence of the EO.

### 3.6. Release Study

**Figure 4:**
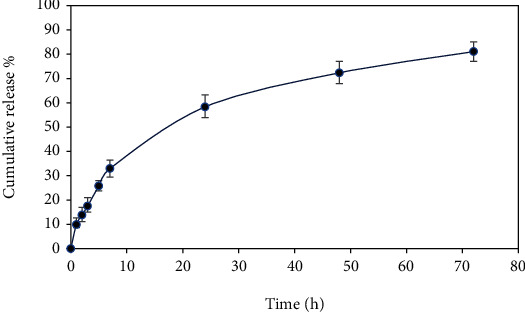
The release profile of *C. copticum L*. EO from the tissue conditioner.

Figure 4 shows the releasing profile of the *C. copticum L*. EO from the tissue conditioner. As this figure suggests, the essential oil was released within 3 days and the ultimate cumulative release was 81%. The figure shows that the release within the first hour was about 9%, whereas 58% of the EO was released in the first 24 hours.

## 4. Discussion

Denture stomatitis is an inflammatory condition that can be developed under dentures. The main cause of this condition is fungal agents, particularly *Candida* species, which are normal oral commensals [33]. Incorporating antifungal agents into the tissue conditioner was found to be a viable option to overcome this condition. In this respect, the gradual release of the antifungal agent might inhibit the fungal growth and subsequent biofilm formation. The current study is aimed at preparing a tissue conditioner incorporated with *C. copticum L*. EO to prevent denture stomatitis. *C. copticum L*. was selected due to its comprehensive antibacterial and antifungal activities [34]. In this study, firstly, the EO was prepared and to determine the composition and main components analyzed using GC-MS. As mentioned, the prepared *C. copticum L*. EO of this study is thymol-based, which is similar to the other studies that extract the EO from the plants in the region of Iran. The EO investigated in the study conducted by Rasooli et al. was found to be composed of thymol (37%), p-cymene (32%), and *γ*-terpinene (27%) as the main components [35]. In another study, the composition of the EO was found to be thymol (45.9%), *γ*-terpinene (20.6%), and o-cymene (19%) [36]. A higher amount of thymol can lead to more antimicrobial activity in the EO as other studies showed [37].

The sensitivity of the EO was evaluated against different bacterial and fungal species using the MIC method. In this approach, the activity of different concentrations of the EO was evaluated to determine the minimum effective concentration. The sensitivity test showed that the prepared EO can perform a comprehensive antimicrobial activity against the fungal and gram-positive and gram-negative bacterial species. The obtained results showed that the MIC of the EO against *C. albicans* is 64 *μ*g mL^−1^, which is comparable with the obtained results of Oroojalian et al. and affirms the significant antimicrobial activity of the EO [38]. These results indicate that the incorporation of EO into the tissue conditioner can lead to suitable biological activities.

In the current study, the GC Soft-Liner (GC Corporation, Tokyo, Japan) was used as a tissue conditioner due to its widespread usage. One of the main obstacles in using EO as an antimicrobial agent is its negative effect on the mechanical properties of the tissue conditioners [39]. The mechanical properties of the tissue conditioner incorporated with different EO concentrations were investigated by a stress relaxation test at different times. The stress relaxation test can evaluate the elastic modulus that is an important property of the tissue conditioner. The obtained results showed that the increase of the EO concentration significantly diminishes the elastic modulus on the first day. This effect is attributed to the disturbance in the gelation of the tissue conditioner, which results in poor cohesion among the polymer chains due to the less entanglement [40]. On the seventh day, however, the results were not significantly different; this can be due to the releasing of the EO and increasing of the elasticity with time. This enhancement is because of the increase of the bond strength due to the water absorption in the tissue conditioner's matrix [41]. Comprehensively, the incorporation of the EO resulted in a significant decrease of elastic modulus in the tissue conditioner, but its effect was not significant on the other days.

The functional groups and components of the EO and tissue conditioner were evaluated by FTIR. The FTIR spectra of the EO corroborate the obtained composition of the GC-MS test that is mainly composed of thymol and p-cymene. The comparison between the FTIR spectra of the tissue conditioners with and without EO showed that the characteristic peaks of the EO can be observed in the EO-loaded one. The slight shifts of the FTIR peaks showed that the EO individually exists in the tissue conditioner's matrix.

The incorporated EO should be released throughout the time the tissue conditioner is placed. Therefore, EO can prohibit the growth of fungal species on the gum and inhibit the following consequences like denture stomatitis. The release study showed that there is no significant burst release occurring as the EO release was low within the first hour. Therefore, EO is well trapped in the tissue conditioner and can be released during the tissue conditioner placement. Comparing the amount of EO release in the first, second, and third days shows that the releasing profile follows a sustained release as there is a significant release on the last day. The ultimate cumulative release of 81% on the third day suggests that this system can successfully apply as a delivery system for the 3-day delivery of the EO. The results of the release study affirm the desirability of this delivery system and its potential to inhibit the growth of microorganisms.

The formation of biofilm on different surfaces can be reported by quantifying the biofilm mass along different time points. Methods such as spectrophotometric analysis, colony-forming counting units (CFU), and colorimetric assays, such as crystal violet, eosin, and XTT reduction assay, have determined the microbial biofilms. However, each method has limitations. For example, CFU count has been shown to underrepresent the cell number. In spectrophotometric analyses of cell density, all cells, both living and dead, contribute to the readings, which produces an overestimation of cell count. The XTT reduction assay depends on cell activity, instead of cell mass, and has been used as a routine method for the quantification of microbial biofilms because it measures cell activity [42–44].

Finally, the biofilm formation was observed to evaluate the activity of the conditioner in the prohibition of fungal and bacterial growth. Scanning electronic microscopy could be used as a semiquantitative technique for fungal micromorphology evaluation, because it allows the observation of microbial-surface interactions. In the present study, the SEM images were taken from the tissue conditioner with and without EO, and these pictures showed that during the formation of blastoconidia, pseudohyphae, and hyphae, particularly formed on the tissue conditioner without the EO, the incorporation of the EO results in the almost total inhibition of biofilm formation, which plays a key role in oral diseases [45]. Thus, in this study, the finding of scanning electron microscopy confirmed the results obtained from the XTT assay. These structures are related to biofilm formation and microbial pathogenicity because it becomes a barrier against phagocytosis. These results were also in line with Gondim et al.'s study [46].

## 5. Conclusion

The results obtained in the present study indicated that the EO of *C. copticum L.* has a considerable antimicrobial activity. These results suggest that the *C. copticum L*. EO-loaded tissue conditioner can be a viable candidate for the simultaneous treatment of the injured mucosa and denture stomatitis. As the industries tend to use natural preservatives instead of chemical additives in their products, the EO of *C. copticum L.* with potential antimicrobial activities might be considered a proper natural source to control microbial contaminations in the products and improve their shelf life and quality. On the other hand, regarding the growing problem of microbial resistance to antimicrobial agents, *C. copticum L*. can also be used for developing novel antimicrobial agents in order to control microbial infections and their biofilm formation.

## Figures and Tables

**Table 1:** The composition of *C. copticum L*. EO obtained by GC-MS.

Peak	Start	RT	End	Area	Area (%)	Cal ki	Compound
1	6.204	6.249	6.318	1719610	0.262044869	922.5197	Thujene (*α*-)
2	6.41	6.448	6.502	472424	0.073556455	930.3543	Pinene (*α*-)
3	7.435	7.474	7.535	2502531	0.381574108	970.748	Pinene (*β*-)
4	7.673	7.711	7.795	1815694	0.275836705	980.0787	Myrcene
5	8.506	8.69	8.759	1.87*E* + 08	28.42956969	1014.135	Cymene (*ρ*-)
6	8.858	8.92	8.981	2681871	0.409157779	1021.053	Sylvestrene
7	9.731	9.884	9.937	1.57*E* + 08	23.85987495	1050.045	Terpinene (*γ*-)
8	10.894	10.955	11.062	584060	0.08734829	1082.256	Terpinolene
9	13.755	13.847	13.939	642814	0.096542847	1126.611	Terpineol (*cis*-*β*)
10	15.92	16.004	16.196	1012222	0.151710188	1215.542	Carvone
11	17.994	18.262	18.292	3.02*E* + 08	45.97278411	1271.781	Thymol

**Table 2:** The MICs of the *C. copticum L.* EO against the bacterial and fungal species strains.

Strains	ATCC/CBS	MIC (*μ*g mL^−1^)	Fluconazole	Ampicillin
*S. mutans*	35668	32	—	8
*S. sobrinus*	27607	128	—	4
*S. sanguinis*	10556	64	—	2
*S. salivarius*	9222	32	—	4
*S. aureus*	25923	64	—	1
*E. faecalis*	51299	128	—	8
*C. albicans*	10261	64	8	—
*C. dubliniensis*	8500	32	2	—

Note: ATCC: American Type Culture Collection; CBS: CentraalBureau voor Schimmelcultures; MIC: minimum inhibitory concentration.

**Table 3:** Biofilm formation of bacterial and fungal standard strains on the tissue conditioner disks containing different concentrations of *C. copticum L.* EO.

Standard strains		Vitality value percent on the tissue conditioner disks with different concentrations of *C. copticum L.* EO				
Strains	ATCC/CBS	0 *μ*g mL^−1^	8 *μ*g mL^−1^	16 *μ*g mL^−1^	32 *μ*g mL^−1^	64 *μ*g mL^−1^
		Vitality (%) ± SD	Vitality (%) ± SD	Vitality (%) ± SD	Vitality (%) ± SD	Vitality (%) ± SD
*S. mutans*	35668	100	63.15 ± 3.2	39.47 ± 2.6	37.89 ± 3.1	36.3 ± 2.5^∗^
*S. sobrinus*	27607	100	90 ± 4.5	83.3 ± 4.8	71 ± 3.6	60 ± 4.5
*S. sanguinis*	10556	100	97.7 ± 5.2	77.7 ± 3.2	66.6 ± 4.1	55.27 ± 2.3
*S. salivarius*	9222	100	48.7 ± 2.9	32.05 ± 2.1^∗^	24.35 ± 2.5	22.43 ± 2.5^∗∗^
*S. aureus*	25923	100	68.1 ± 3.2	60.0 ± 3.2	58.18 ± 3.3	48.18 ± 4.1
*E. faecalis*	51299	100	88.75 ± 2.5	65.0 ± 2.9	78.75 ± 5.1	62.5 ± 5.5
*C. albicans*	10261	100	88.5 ± 4.5	85.7 ± 5.1	76.19 ± 3.1	71.42 ± 2.6
*C. dubliniensis*	8500	100	62.5 ± 3.6	50.0 ± 4.5	49.37 ± 2.6	43.75 ± 3.6

Note: ATCC: American Type Culture Collection; CBS: CentraalBureau voor Schimmelcultures; SD: standard deviation. ^∗^*P* value < 0.05; ^∗∗^*P* value ≤ 0.001.

## Data Availability

The data used to support the findings of this study were supplied by Shiraz University of Medical Sciences under license and so cannot be made freely available. Requests for access to these data should be made to Kamiar Zomorodian, zomorodian@sums.ac.ir or kzomorodian@gmail.com.
